# Epidemiological and Histopathological Characteristics of Fetuses with Congenital Disorders: A Study in Greece

**DOI:** 10.3390/biology14060626

**Published:** 2025-05-29

**Authors:** Despoina Nteli, Maria Nteli, Konstantinos Konstantinidis, Maria Ouzounidou, Paschalis Theotokis, Maria-Eleni Manthou, Iasonas Dermitzakis, Xeni Miliara, Chrysoula Gouta, Stamatia Angelidou, Dimosthenis Miliaras, Soultana Meditskou

**Affiliations:** 1 Laboratory of Histology and Embryology, School of Medicine, Faculty of Health Sciences, Aristotle University of Thessaloniki, 54124 Thessaloniki, Greece; despntel@gapps.auth.gr (D.N.); marintel@gapps.auth.gr (M.N.); konstantink@auth.gr (K.K.); mariaouzou@auth.gr (M.O.); ptheotokis@gmail.com (P.T.); mmanthou@auth.gr (M.-E.M.); iasonasd@auth.gr (I.D.); miliaras@auth.gr (D.M.); 2 Histopathology Laboratory, Euromedica General Clinic of Thessaloniki, 6 Thalitos Street, 54645 Thessaloniki, Greece; xmiliara@gmail.com; 3 Department of Pathology, Hippokration General Hospital of Thessaloniki, Konstantinoupoleos 49, 54642 Thessaloniki, Greece; ch.gouta@gmail.com (C.G.); dpalab@ippokratio.gr (S.A.)

**Keywords:** congenital disorders, risk factors, Greece, epidemiology, cross-sectional study

## Abstract

Congenital malformations constitute a major public health issue. Nonetheless, Greece does not participate in the European network of congenital anomalies. To examine the epidemiology of congenital abnormalities and investigate possible factors associated with their development, autopsies were performed on 649 fetuses referred from three healthcare facilities in Thessaloniki during 1992–2008. Adequate statistical analysis methods were implemented. Birth defects were found in 256 fetuses (39.5%)—primarily related to the musculoskeletal (17.3%), nervous (14.5%), cardiovascular (12.5%) and urinary (10.4%) systems. A statistically significant positive correlation (*p*-values < 0.05) emerged between the presence of a congenital defect and intrauterine growth restriction, inability to identify the fetus’ sex, iatrogenic abortion, nuchal oedema and a single umbilical artery. An inverse association (*p*-values < 0.05) was deduced with the gestational age, twin pregnancy, stillbirth, chorioamnionitis, infarction and intervillous thrombus of the placenta and nuchal cord. In multivariable analysis, the relationship remained significant (*p*-values < 0.05) between the development of birth defects and iatrogenic abortion, nuchal oedema, presence of a single umbilical artery, chorioamnionitis, infarction of the placenta and nuchal cord. ROC curve analysis indicated area under the curve = 0.800, 95% confidence interval = 0.76–0.84, *p*-value = 0.000. Our study revealed the epidemiology of congenital anomalies in Greece, confirming the relationship between various factors and birth defect occurrence.

## Simple Summary

Birth defects pose a significant public health problem. However, Greece is not a part of the European network of congenital anomalies and no recent studies examining congenital malformations have been carried out in this country. The aim of the present work is to study the epidemiology of birth defects in Greece and identify possible factors associated with their occurrence. The study population consisted of 649 fetuses that were referred from three hospitals in Thessaloniki for autopsy and histo-pathological examination over a period of 16 years (1992–2008). Congenital anomalies were identified in 256 out of the 649 fetuses and were primarily associated with the musculoskeletal, nervous, cardiovascular and urinary systems. Various factors were found to be statistically significantly related—both positively and negatively—to the development of congenital anomalies in univariable and multivariable logistic regression analysis. Our autopsy-based study revealed the epidemiology of congenital anomalies in Northern Greece and corroborated the relationship between a number of factors and birth defect occurrence.

## 1. Introduction

Congenital disorders, or congenital malformations, are defined as structural and functional deviations from normal development that are present at birth and are established during intrauterine life [1]. They may occur as single disease entities or as part of other syndromes, affecting one or more organ systems [2].

The aetiology of congenital disorders, although not always clear, involves both genetic factors and a variety of environmental influences, including maternal infections and diseases, maternal diet and exposure to harmful substances, such as tobacco, alcohol and chemicals [3].

Birth defects constitute a major public health issue, since they have been associated with increased morbidity and mortality in fetuses and newborns/infants, entail significant costs for the medical care of affected individuals and are associated with multiple negative consequences for the quality of life and well-being of both the patients and their families [3,4].

Congenital abnormalities, annually, occur in 2.5% of all births in Europe [5]. Their incidence does not seem to decrease over time [6]. Consequently, careful epidemiological surveillance is necessary to identify possible causative factors and to implement appropriate preventive measures.

The present paper aims to examine the epidemiology of birth defects in Northern Greece, as there is a lack of studies on the prevalence of congenital abnormalities in Greece. To the best of our knowledge, no other recent study with a similar purpose has been conducted in Greece, which does not participate in the European network of congenital anomalies (EUROCAT—European Surveillance of Congenital Anomalies). Additionally, in an attempt to contribute to the existing scientific literature, we investigated possible factors associated with the occurrence of congenital malformations using data from autopsies of fetuses and newborns from the region of Northern Greece.

## 2. Materials and Methods

### 2.1. Study Population and Data Sources

The study population consisted of 649 fetuses and newborns who were referred to the Histopathological Laboratory of Hippokration General Hospital of Thessaloniki and the private Histopathological Laboratory ‘ISTOTYPOS IKE’ for autopsy and histopathological examination over a period of 16 years (1992–2008). All fetuses originated from stillbirths or iatrogenic abortions and were derived from three healthcare facilities in Thessaloniki (Hippokration General Hospital of Thessaloniki, GENESIS Obstetrics Gynaecology Surgery Hospital and Euromedica General Clinic of Thessaloniki).

Iatrogenic abortions were performed following the detection of abnormal ultrasound findings. The abortion techniques varied among the healthcare facilities. No detailed report was provided to the pathologist performing the autopsies and the histopathological examinations. In general, standard methods, including medications and/or surgical procedures, were followed depending on the gestational age and preferences of the mother and the attending physician. In Greece, abortion is permitted during the first 12 weeks of pregnancy, but this period of time is extended to the following: a. the first 19 weeks in cases of pregnancy resulting from rape, seduction of a minor, incest or abuse of a woman incapable of resisting; b. the first 24 weeks if there are indications of a serious fetal abnormality; c. no time restriction applies if there is an unavoidable risk to the life of the pregnant woman or a risk of serious and lasting damage to her physical or mental health verified by a relevant physician (Article 304 of the Greek Penal Code, as amended by Law 1609/1986).

The key steps of all fetal autopsies included a thorough external examination, which involved assessing fetal weight, crown–heel length and external features, such as dysmorphic signs, skin appearance and visible anomalies. Particular attention was given to identifying signs of generalised or localised oedema and maceration, when present. The topography, morphology, dimensions and weight of all organs within the cranial, thoracic, peritoneal and pelvic cavities were checked, and tissue samples for histological evaluation were taken. The placenta and umbilical cord were also examined for pathological changes, including abnormalities in insertion, vessel number, thrombosis or infarction.

This was followed by histological evaluation of the placenta, membranes, umbilical cord and all internal organs -including the brain, thoracic, abdominal and pelvic structures- with the aim of assessing organ development, detecting structural anomalies and identifying histological evidence of inflammation, infection, hypoxia or other findings. Tissue samples were collected for microscopic observation using standard protocols. In cases where macroscopic pathology or dysmorphic features were detected, additional sections were obtained from the affected organs or regions to ensure a more detailed histopathological assessment.

All clinical and demographic data, along with the main findings of the histopathological reports, were stored in an electronic database. Data were sorted by categories (demographics, autopsy and histopathological findings of fetuses and extraembryonic tissues, identified congenital anomalies).

### 2.2. Outcomes

The presence of congenital anomalies as well as the specific organ systems from which they originated was identified during the autopsy performed on each of the studied fetuses.

### 2.3. Exposures

Exposures of the research participants were assessed based on the conclusions of the post-mortem and histologic examinations. We categorised the findings of the fetuses and their extraembryonic tissues as follows: (a) demographic features of each fetus and its mother (mother’s age, gestational age, sex of the fetus, fetal weight, presence of twin pregnancy, type of abortion/delivery) and (b) autopsy and histopathological findings of the examined fetuses and their extraembryonic tissues (detection of nuchal oedema, chorioamnionitis, placental anomalies, umbilical cord aberrations or intrauterine growth restriction).

### 2.4. Statistical Analysis

Depending on the existence of a normal or non-normal distribution, continuous variables were reported using the mean and standard deviation (SD) or the median and interquartile range (IQR), respectively. Between-group comparisons (fetuses with congenital anomalies vs. fetuses without congenital anomalies) were conducted utilising the Student’s *t*-test for normally distributed continuous variables and the Mann–Whitney U test for those with non-normal distribution. Categorical variables were reported as frequencies (%) and a Pearson’s chi-squared test or Fisher’s exact test was performed for between-group comparisons. Univariable and multivariable logistic regression analysis and receiver operating characteristic (ROC) curve analysis were used to identify predictors of the development of congenital anomalies and to assess their predictive ability. A *p*-value < 0.05 was considered statistically significant. Data management and statistical analyses were performed using SPSS software version 28 (IBM Corp., Armonk, NY, USA). GraphPad Prism 10.1.1 (GraphPad Software, Boston, MA, USA) and Microsoft Power BI Desktop (version 2.143.878.0) were used for graph creation.

### 2.5. Ethics Approval

The study was conducted in compliance with the Declaration of Helsinki and was approved by the Ethics Committee of the School of Medicine, Aristotle University of Thessaloniki (number of approval: 6595, date of approval: 14 June 2022). Informed consent was obtained by the mother or both parents of each fetus.

## 3. Results

**Figure 1:**
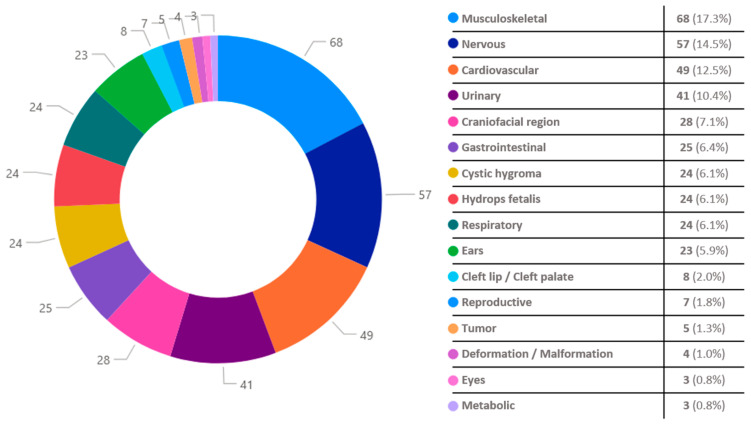
Congenital disorders by organ system/type of anomaly [number of fetuses with the identified anomaly (percentage % of all the recorded anomalies)].

A total of 649 fetuses were examined both macroscopically and microscopically and congenital disorders were identified in 256 cases (39.5%). Co-existence of multiple birth defects was detected in 97 fetuses (15.0%). In total, 393 anomalies were recorded. The most frequently affected systems were the musculoskeletal (68 out of the 256 fetuses—17.3% of the 393 anomalies), nervous (57 out of 256—14.5%), cardiovascular (49 out of 256—12.5%) and urinary systems (41 out of 256—10.4%) [Figure 1]. The most commonly detected abnormality in the musculoskeletal system was clubfoot; in the nervous system, hydrocephalus; in the cardiovascular system, ventricular septal defect; and in the urinary system, renal agenesis. Demographics and histopathological findings of the examined fetuses are presented in Table 1. 

**Figure 2:**
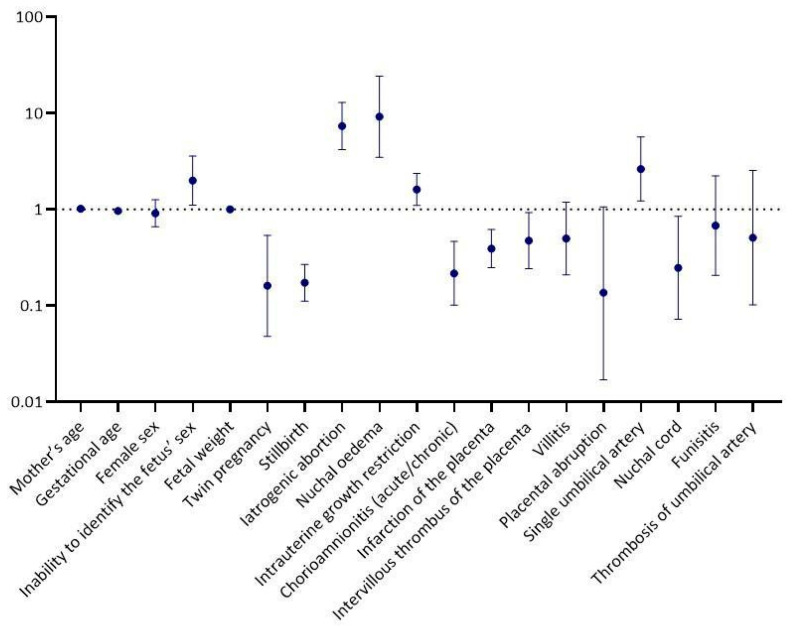
Independent predictors of congenital disorder development—univariable logistic regression analysis.

The univariable logistic regression analysis revealed a statistically significant positive correlation between congenital disorders and the following factors: intrauterine growth restriction (odds ratio—OR = 1.61, 95% confidence interval—CI = 1.10, 2.36, *p* = 0.014), inability to identify the sex of the fetus (OR = 1.99, 95% CI = 1.11, 3.57, *p* = 0.022), iatrogenic abortion (OR = 7.35, 95% CI = 4.18, 12.93, *p* < 0.001), presence of nuchal oedema (OR = 9.15, 95% CI = 3.48, 24.09, *p* < 0.001) and a single umbilical artery (OR = 2.63, 95% CI = 1.22, 5.66, *p* = 0.014). In contrast, a statistically significant negative association was found between the occurrence of congenital abnormalities and the following factors: gestational age (OR = 0.97, 95% CI = 0.94, 0.99, *p* = 0.002), twin pregnancy (OR = 0.16, 95% CI = 0.05, 0.54, *p* = 0.003), stillbirth (OR = 0.17, 95% CI = 0.11, 0.27, *p* < 0.001), chorioamnionitis (acute/chronic) (OR = 0.22, 95% CI = 0.10, 0.46, *p* < 0.001), infarction of the placenta (OR = 0.39, 95% CI = 0.25, 0.62, *p* < 0.001), intervillous thrombus of the placenta (OR = 0.47, 95% CI = 0.24, 0.93, *p* = 0.029) and nuchal cord (OR = 0.25, 95% CI = 0.07, 0.85, *p* = 0.026) [Figure 2 and Table 2].

**Figure 3:**
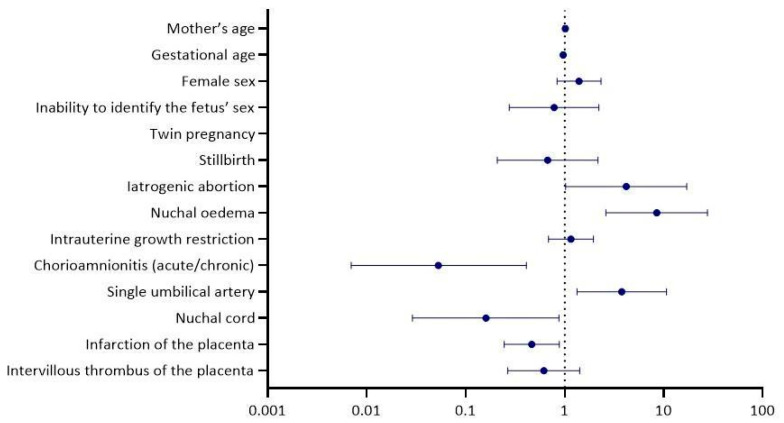
Independent predictors of congenital disorder development—multivariable logistic regression analysis.

**Figure 4:**
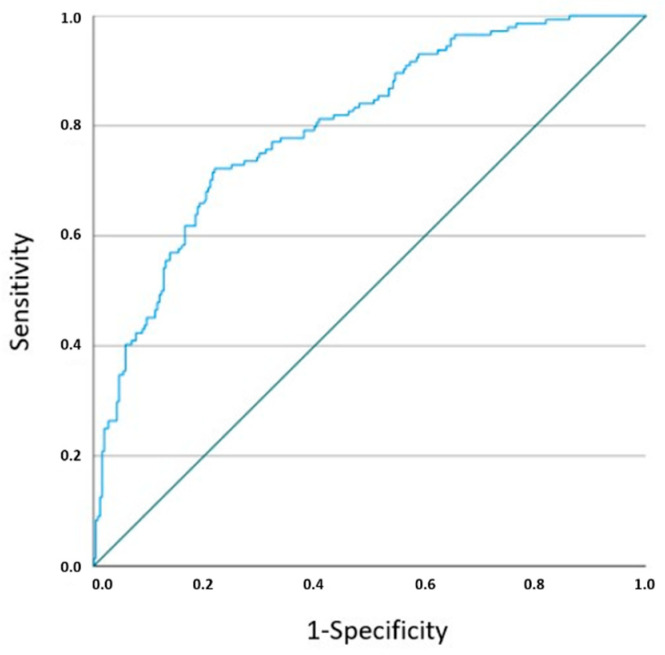
Receiver operating characteristic (ROC) curve analysis on the predictive ability of the created multivariable model. The diagonal green line (sensitivity = 1 − specificity) represents the line of no discrimination, indicating the performance of a classifier that makes random predictions.

Variables identified as statistically significant in the univariable regression analysis, along with other clinically important variables, were included in the multivariable model [Table 2 and Figure 3]. In the multivariable regression analysis, the relationship remained statistically significant between the development of birth defects and iatrogenic abortion (adjusted OR-aOR = 4.18, 95% CI = 1.02, 17.10, *p* = 0.046), nuchal οedema (aOR = 8.49, 95% CI = 2.61, 27.66, *p* < 0.001), a single umbilical artery (aOR = 3.77, 95% CI = 1.33, 10.66, *p* = 0.012), chorioamnionitis (acute/chronic) (aOR = 0.05, 95% CI = 0.01, 0.41, *p* = 0.005), infarction of the placenta (aOR = 0.47, 95% CI = 0.25, 0.88, *p* = 0.019) and nuchal cord (aOR = 0.16, 95% CI = 0.03, 0.88, *p* = 0.035). ROC curve analysis for the developed multivariable model yielded an area under the curve—AUC = 0.800, 95% CI = 0.76, 0.84, *p* = 0.000 [Figure 4].

## 4. Discussion

The present retrospective study aims to examine the epidemiology of congenital anomalies in Northern Greece and investigate factors associated with their occurrence. The main organ systems in which most congenital anomalies were detected were the following: musculoskeletal system, nervous system, cardiovascular system, urogenital system, craniofacial region and gastrointestinal system. Additionally, after adjustment for univariably significant and other clinically important variables, a statistically significant correlation emerged between the occurrence of congenital anomalies and the presence of iatrogenic abortion, nuchal oedema, a single umbilical artery, chorioamnionitis, infarction of the placenta and nuchal cord.

In general, regarding the organ systems with the highest frequency of congenital abnormalities, our findings were in line with the results of other studies [3]. In particular, almost identical results have been reported by the EUROCAT network of population-based registries for the epidemiological surveillance of congenital anomalies, which has monitored fetuses with congenital abnormalities originating from iatrogenic abortions or intrauterine fetal demise since 1979. According to their data, in the cases without chromosomal abnormalities, the most commonly affected systems, in order of frequency, were as follows: cardiovascular system, limbs, urinary system and nervous system [3,5].

Similarly, Türkbay et al., who analysed data from 102,379 live births over a six-year period, concluded that the most common abnormalities were congenital heart anomalies, Down syndrome and meningomyelocele [7]. A meta-analysis of 25 studies investigating the prevalence of birth defects in sub-Saharan African countries demonstrated that musculoskeletal anomalies, followed by cardiovascular disorders, gastrointestinal abnormalities, orofacial clefts, urogenital defects and Down syndrome constituted the most frequently observed deviations from normal development [8]. In another multi-year study from the US, Aggarwal et al. found that the majority of congenital abnormalities were, in order of frequency, associated with the cardiovascular system, gastrointestinal system, eyes, musculoskeletal system, ears, urogenital system and craniofacial region (cleft lip/cleft palate) [9].

It is worth mentioning that some researchers reported chromosomal abnormalities as the most frequent type of congenital disorder [9], a finding that we were not able to verify in the present study due to significant inconsistencies between the histopathological reports regarding whether karyotyping had been performed.

Longer gestational duration was associated with a reduced risk of congenital malformations, which is consistent with previously published studies, suggesting that the incidence of congenital anomalies is up to more than twice as high in preterm compared to full-term births, with the risk increasing as the duration of intrauterine life decreases [10,11]. More specifically, Honein et al., who analysed data from 13 American States, found that very preterm neonates (24–31 weeks) exhibited a more than fivefold higher prevalence of congenital abnormalities, primarily affecting the central nervous and cardiovascular systems, compared to full-term neonates [11]. In another study, a gestational age of ≤36 weeks was identified as an independent risk factor for congenital disorders [12].

In compliance with the existing literature, we detected positive associations between the presence of congenital malformations and the following factors: intrauterine growth restriction, nuchal oedema and a single umbilical artery.

According to some studies, intrauterine growth restriction has been associated with an increased likelihood of certain congenital abnormalities [13,14]. However, other studies have failed to demonstrate a similar statistically significant correlation [15], although they do highlight a higher incidence of congenital disorders in fetuses with asymmetric growth restriction compared to those with symmetric growth restriction [15,16].

Regarding increased nuchal translucency, Guo et al. concluded that it is related to higher rates of adverse outcomes, including spontaneous abortion, as well as to a wide range of structural anomalies (such as defects of the nervous system, cardiac and musculoskeletal system) and genetic syndromes (e.g., trisomy 21, 18, 13, 9 and sex chromosome abnormalities) [17]. The association between increased nuchal translucency and the occurrence of congenital heart defects has also been supported by other researchers [18]. Additionally, Bellai-Dussault et al. found that a nuchal translucency measurement <2 mm was associated with the lowest risk of chromosomal abnormalities and emphasized that the risk of anomalies increases with greater nuchal translucency, potentially warranting further testing even when measurements fall below the standard 3.5 mm cutoff [19]. Finally, greater nuchal translucency has been linked to adverse pregnancy outcomes, including fetal anomalies, hydrops fetalis and preterm labour, even in cases where no chromosomal abnormalities were identified by karyotyping [20].

The presence of a single umbilical artery is consistently associated with an increased risk of chromosomal and other congenital abnormalities—a finding also verified in the present study. Ebbing et al. reported that a single umbilical artery was identified in 11.0% of the studied fetuses with birth defects, with particularly strong associations observed for gastrointestinal atresia/stenosis, nasal agenesis, cardiovascular abnormalities (7–8 times increased risk) and central nervous system disorders [21]. Ju et al. concluded that 39.9% of fetuses with a single umbilical artery presented with structural abnormalities, most commonly oesophageal stenosis or atresia, followed by cardiovascular, scoliosis, genitourinary and brain malformations [22]. The study of Varafai et al. demonstrated that the presence of a single umbilical artery displayed a statistically significant correlation with renal, cardiac and other congenital abnormalities, as well as with adverse perinatal outcomes including intrauterine fetal death, early neonatal death, low birth weight and prematurity (OR = 68.02, 31.04, 16.03, 3.85, 11.31, 3.22, 2.47, respectively) [23]. Finally, Saxena et al. noted that urinary tract, lung and musculoskeletal abnormalities constituted the predominant types of birth defects occurring in fetuses with a single umbilical artery [24].

Surprisingly, we found that twin pregnancy and the presence of chorioamnionitis were negatively associated with the likelihood of congenital disorder development. This result contradicts the existing literature, in which an increased incidence of congenital abnormalities in twins in comparison with singletons as well as a more frequent occurrence of birth defects in case of chorioamnionitis have been reported. Dawson et al. detected a significant positive correlation between twin pregnancy and 29 of the 45 congenital abnormalities examined [25], a finding that was also verified by Ting et al., who reported a statistically significant difference in the rate of birth defects between twins and singletons [26]. Additionally, both the studies of Gijtenbeek et al. and Best et al. demonstrated a significantly increased risk of certain congenital heart defects in twin pregnancies compared to single pregnancies [27,28]. Regarding chorioamnionitis, most research papers stated that clinical chorioamnionitis constituted an important cause of perinatal mortality and long-term morbidity, as it was associated with decreased cognitive and motor development in affected infants [29,30,31,32].

Moving on, we discovered an inverse relationship between the presence of a nuchal cord as well as certain placental pathologies (intervillous thrombus, infarction) and the occurrence of congenital abnormalities—findings that are not sufficiently supported by the current literature. The nuchal cord has been mainly associated with intrauterine death, preterm delivery, perinatal hypoxia/asphyxia and a low 1-min Apgar score [33,34,35,36]. Meanwhile, placental ischemia and placental intervillous thrombi were found to be linked to an increased risk of specific congenital abnormalities (e.g., birth defects of the cardiovascular and central nervous system, craniofacial abnormalities, congenital disorders of the limbs) [37,38] and adverse pregnancy outcomes (e.g., higher incidence of intrauterine growth restriction, placental abruption, preterm delivery) [39,40], respectively.

Additionally, we should acknowledge the fact that our study did not unveil an association between congenital malformations and maternal age. However, according to data from other research papers, maternal age <20 and >40 is related to a higher risk of specific congenital abnormalities [41,42,43]. Similar results were reported in a meta-analysis by Ahn et al., which nonetheless noted that the quality of the data used to draw conclusions was low, thus the findings should be interpreted with caution [44].

According to the literature, male fetuses appear to exhibit a higher prevalence of certain congenital abnormalities [45,46,47,48]. However, our work did not reveal a statistically significant difference in the occurrence of congenital disorders between male and female fetuses. Instead, we found that the presence of birth defects was primarily associated with the inability to determine the sex of the fetus -predominantly in early preterm infants- rather than with fetal sex per se.

### 4.1. Strengths of the Study

We conducted a multicenter study with data collected from three major healthcare facilities in Northern Greece. The Obstetrics Departments involved manage a substantial volume of cases, which enhances the representativeness of our study population and ensures a more than adequate sample size. Furthermore, our study spans a considerable period, covering the years 1992–2008.

### 4.2. Limitations of the Data

Certain factors pose constraints on the generalisability of this paper’s conclusions. These limitations can be attributed to the fact that this is a retrospective observational study, inevitably vulnerable to errors and unable to prove cause-and-effect relationships. In addition to this, the use of data from a relatively old cohort (1992–2008) may affect the applicability of our findings to current clinical practice, thereby necessitating further research as far as the trends of congenital anomalies in the years between 2008 and 2025 are concerned. Moreover, the data throughout the years were heterogeneously registered and karyotyping for the detection of chromosomal abnormalities was performed selectively rather than systematically, thus we were unable to draw reliable conclusions regarding the prevalence of chromosomal anomalies. Finally, unrecognised residual confounding could still affect our results in spite of our attempt to minimise confounding factors by using multivariable adjustment.

## 5. Conclusions

In conclusion, the present study provides a comprehensive statistical analysis of the epidemiological and embryological characteristics of fetuses and newborns with congenital disorders in Northern Greece. The predictive multivariable model we designed confirmed the significant association of nuchal oedema and a single umbilical artery with the occurrence of birth defects and demonstrated satisfactory predictive performance. In any case, further studies, utilising high-quality data from well-organised registries, are needed.

## Figures and Tables

**Table 1:** Demographics and histopathological findings of the studied fetuses.

	Total Fetuses (N = 649)	Fetuses with Congenital Disorders (N = 256)	Fetuses Without Congenital Disorders (N = 393)	*p*-Value
**Demographics**				
Mother’s age (years) *	29.86 (5.653)	30.10 (5.756)	29.70 (5.590)	0.205
Gestational age (weeks) *	23.00 (12.00)	22.00 (7.00)	23.00 (14.00)	**0.020**
Sex				
Male	335 (51.6)	128 (50.0)	207 (52.7)	0.506
Female	265 (40.8)	101 (39.5)	164 (41.7)	0.564
Cannot be identified ^+^	49 (7.6)	27 (10.5)	22 (5.6)	**0.020**
Fetal weight (grams) *	540.00 (1227)	530.00 (934)	543.50 (1407)	0.665
Twin pregnancy	30 (4.6)	3 (1.2)	27 (6.9)	**<0.001**
Type of abortion/delivery				
Stillbirth	518 (80.9)	160 (64.5)	358 (91.3)	**<0.001**
Iatrogenic	79 (12.3)	62 (25.0)	17 (4.3)	**<0.001**
Vaginal delivery/C-section	43 (6.7)	26 (10.5)	17 (4.3)	**0.002**
**Autopsy and histopathological findings of fetuses and extraembryonic tissues**				
Nuchal oedema	32 (4.9)	27 (10.5)	5 (1.3)	**<0.001**
Intrauterine growth restriction	174 (36.2)	78 (43.1)	96 (32.0)	**0.014**
Chorioamnionitis (acute/chronic)	59 (9.1)	8 (3.1)	51 (13.0)	**<0.001**
Placenta				
Infarction	122 (18.8)	28 (10.9)	94 (23.9)	**<0.001**
Intervillous thrombus	49 (7.6)	12 (4.7)	37 (9.4)	**0.026**
Villitis	28 (4.3)	7 (2.7)	21 (5.3)	0.110
Abruption	12 (1.8)	1 (0.4)	11 (2.8)	**0.034**
Umbilical cord				
Single umbilical artery	29 (4.5)	18 (7.0)	11 (2.8)	**0.011**
Nuchal cord	21 (3.2)	3 (1.2)	18 (4.6)	**0.016**
Funisitis	13 (2.0)	4 (1.6)	9 (2.3)	0.518
Thrombosis of umbilical artery	8 (1.2)	2 (0.8)	6 (1.5)	0.490

Continuous variables (marked with an asterisk) are described using means and standard deviations (in the parentheses) in case of a normal distribution or with medians and interquartile ranges (in the parentheses) in case of a non-normal distribution. Categorical variables are described by the number of fetuses presenting with each of the listed characteristics and the percentage (in the parenthesis). Statistically significant values (in bold) are considered the ones with a *p*-value < 0.05. Inability to identify the sex of an embryo (marked with a +) can be attributed to it being at an early gestational age or its gonads having undergone autolysis.

**Table 2:** Independent predictors of congenital disorder development—univariable and multivariable logistic regression analysis.

	Univariable Logistic Regression Analysis	
	*p*-Value	OR ^a^ (95% CI ^b^)
Mother’s age	0.409	1.01 (0.98, 1.04)
Gestational age	**0.002**	0.97 (0.94, 0.99)
Female sex	0.564	0.91 (0.66, 1.25)
Inability to identify the fetus’ sex	**0.022**	1.99 (1.11, 3.57)
Fetal weight	0.187	1.00 (1.00, 1.00)
Twin pregnancy	**0.003**	0.16 (0.05, 0.54)
Stillbirth	**<0.001**	0.17 (0.11, 0.27)
Iatrogenic abortion	**<0.001**	7.35 (4.18, 12.93)
Nuchal οedema	**<0.001**	9.15 (3.48, 24.09)
Intrauterine growth restriction	**0.014**	1.61 (1.10, 2.36)
Chorioamnionitis (acute/chronic)	**<0.001**	0.22 (0.10, 0.46)
Infarction of the placenta	**<0.001**	0.39 (0.25, 0.62)
Intervillous thrombus of the placenta	**0.029**	0.47 (0.24, 0.93)
Villitis	0.116	0.50 (0.21, 1.19)
Placental abruption	0.057	0.14 (0.02, 1.06)
Single umbilical artery	**0.014**	2.63 (1.22, 5.66)
Nuchal cord	**0.026**	0.25 (0.07, 0.85)
Funisitis	0.520	0.68 (0.21, 2.22)
Thrombosis of umbilical artery	0.409	0.51 (0.10, 2.54)
	**Multivariable logistic regression analysis**	
	***p*-value**	**OR ^a^ (95% CI ^b^)**
Mother’s age	0.548	1.01 (0.97, 1.06)
Gestational age	0.051	0.97 (0.94, 1.00)
Female sex	0.199	1.40 (0.84, 2.33)
Inability to identify the fetus’ sex	0.642	0.78 (0.28, 2.22)
Twin pregnancy	0.998	0.00 (0.00, 0.00)
Stillbirth	0.506	0.67 (0.21, 2.17)
Iatrogenic abortion	**0.046**	4.18 (1.02, 17.10)
Nuchal oedema	**<0.001**	8.49 (2.61, 27.66)
Intrauterine growth restriction	0.583	1.16 (0.69, 1.95)
Chorioamnionitis (acute/chronic)	**0.005**	0.05 (0.01, 0.41)
Single umbilical artery	**0.012**	3.77 (1.33, 10.66)
Nuchal cord	**0.035**	0.16 (0.03, 0.88)
Infarction of the placenta	**0.019**	0.47 (0.25, 0.88)
Intervillous thrombus of the placenta	0.254	0.62 (0.27, 1.42)

^a^ OR = odds ratio, ^b^ 95% CI = 95% confidence interval, statistically significant values (in bold) are considered the ones with a *p*-value < 0.05.

## Data Availability

The data utilised for the present study are available upon request from the corresponding author (Soultana Meditskou, e-mail: sefthym@auth.gr).
